# Association between Serum Copper, Selenium, Zinc, and Serum Estradiol in Women

**DOI:** 10.1155/2022/8747693

**Published:** 2022-09-15

**Authors:** Qianqian Liang

**Affiliations:** Division of Health Management, Yuncheng Central Hospital, Eighth Clinical Medical College of ShanXi Medical University, Yun Cheng, Shanxi 044000, China

## Abstract

**Objectives:**

The objective is to examine the associations between serum copper, selenium, zinc, and serum estradiol (E2) among 2388 female participants from the National Health and Nutrition Examination Survey (NHANES).

**Methods:**

To estimate the association between serum copper, selenium, zinc, and serum E2, multivariate logistic regression analyses were conducted. Fitted smoothing curves and generalized additive models were also performed.

**Results:**

After adjusting for confounding factors, it was found that female serum copper was positively correlated with serum E2. The relationship between serum copper and E2 showed an inverted U-shaped curve (inflection point: 28.57 *μ*mol/L). Serum selenium in women was negatively correlated with serum E2, and in the subgroup of women aged 25-55, the relationship between serum selenium and E2 showed an inverted U-shaped curve (inflection point: 1.39 *μ*mol/L). There was no correlation between serum zinc and serum E2 in women.

**Conclusions:**

Our study revealed a correlation between serum copper and selenium and serum E2 in women and identified an inflection point for each.

## 1. Introduction

Ovarian steroid hormones, including androgens and estrogens, affect the process of folliculogenesis and oogenesis by interacting with specific receptors [1]. Serum estradiol (E2) and follicle size are routine parameters used to monitor follicle development and oocyte maturation [2]. Naoko Kubo's research team found that the use of E2 during growth culture enhanced porcine oocyte meiotic and developmental capacity, cumulus expansion capacity, and cumulus cellperformeattachment [3]. Nayara López Carpintero's research team found that compared with fertilization failure, grade B embryos from oocytes soaked in follicular fluid had higher E2 values and E2/progesterone (P4) and E2/testosterone ratios than grade C embryos [4]. These scholars' studies have shown that E2 plays an important role in the development of follicles; however, the factors that affect changes in E2 levels have not been fully studied.

Minerals are inorganic elements necessary for vital functions in human life. The most abundant elements are carbon, hydrogen, oxygen, and nitrogen. Although they are not classified as nutritional minerals, they make up approximately 96% of the body weight, while macrominerals (i.e., magnesium, sodium, calcium, sulfur, chlorine, phosphorus, and potassium) and trace minerals (i.e., manganese, zinc, cobalt, copper, molybdenum, fluoride, iron, iodine, and selenium) constitute the remaining 4% [5]. Many scholars have studied the role of micronutrients (minerals and vitamins) in the life cycle from the fertilized egg to aging, including dietary micronutrient supplementation, and have demonstrated the importance of a balanced intake of these micronutrients [6–8]. Yet, it is not clear if micronutrient levels correlate with E2 levels.

Copper is an essential element in living organisms and appears to be involved in estrogenic action [9]. Chen Yiqin's research team analyzed human ovarian granulosa cells that were treated with various concentrations of copper for 24 hours. The 17-E2 levels were increased significantly in the 1.0 and 2.0 *μ*g/mL groups after treatment [10]. The effects of copper and selenium on the activation of the E2 synthesis pathway have been reported [11, 12]. Nikhil Kumar Tej et al. research team found that copper, selenium, and copper + selenium did not affect E2 concentrations in goat granulosa cells, and there was no significant difference in E2 concentrations between groups [13]. Zinc is also an important trace element for human reproductive health, especially in men, and is necessary for the normal functioning of the reproductive system and the process of spermatogenesis. Messersmith EM's research team found an inverse correlation between serum zinc concentration and serum testosterone and E2 levels in men [14].

Although these scholars have performed related research on copper, selenium, zinc, and E2, they all rely on animal research and studies with small samples. Therefore, we used the 2013-2016 National Health and Nutrition Examination Survey (NHANES) data to analyze serum copper, selenium, and zinc levels in women and their correlation with E2 levels.

## 2. Materials and Methods

### 2.1. Statement of Ethics

The study was approved by the Ethics Committee of the National Center for Health Statistics Review Board, and written consent was obtained from each participant. We followed the research methodology used in the article published by Zhongxin Zhu's team [15] to conduct this study: Association between Serum Copper, Selenium, Zinc, and Serum E2 in Women.

### 2.2. Study Population

**Figure 1:**
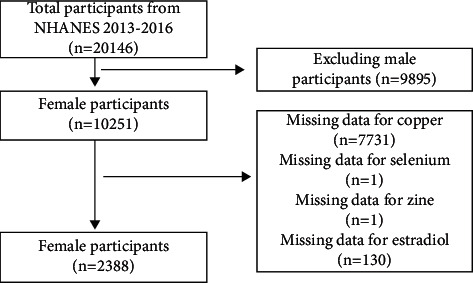
Flow chart of sample selection from the NHANES 2013-2016.

The National Health and Nutrition Examination Survey (NHANES) provides objective data on the health status of children and adults in the United States (US). The NHANES was a large, ongoing cross-sectional survey designed to be nationally representative. In this study, we aggregated data from NHANES for 2 biennial cycles during 2013-2016. Of the 20,146 participants, after excluding male participants (*n* = 9895), missing data for copper (*n* = 7731), missing data for selenium (*n* = 1), missing data for zinc (*n* = 1), and missing data for E2 (*n* = 130), a total of 2388 female participants were included in our analyses (Figure 1).

### 2.3. Variables

The exposure variables in this study were copper, selenium, and zinc. Between 2013 and 2016, copper was measured using inductively coupled plasma dynamic reaction cell mass spectrometry (ICP-DRC-MS). The outcome variable was E2, which was measured by isotope dilution liquid chromatography tandem mass spectrometry (ID-LC–MS/MS). The following categorical auxiliary variables were included in our analysis: race/ethnicity, education level, marriage, poverty-to-income ratio, and body mass index. The continuous covariates included in our analysis were age and total testosterone. Details on copper, selenium, zinc, E2, and the covariates are publicly available at http://www.cdc.gov/nchs/nhanes/.

### 2.4. Statistical Analysis

We performed weighted and variance estimation analyses to account for the marked variance in our dataset. A weighted multivariate logistic regression model was used to evaluate the association between copper, selenium, zinc, and E2. We used the weighted *χ*2 test for categorical variables and the weighted linear regression model for continuous variables to calculate the difference among each group. Subgroup analyses were performed using stratified multivariate regression analysis. Furthermore, smooth curve fittings and generalized additive models were used to address the nonlinear relationship between copper, selenium, and E2. For nonlinear models, the inflection point in the relationship between copper, selenium, and E2 was calculated using a recursive algorithm, with a two-piecewise linear regression model conducted on both sides of the inflection point when nonlinearity was detected. All analyses were performed with the packages *R* (http://www.R.project.org) and EmpowerStats (http://www.empowerstats. com), with a *P* value < 0.05 considered to be statistically significant.

## 3. Results

### 3.1. Levels of Serum E2 Vary in Some Demographic and Health Care Visit Characteristics

A total of 2388 female participants were included in our analyses and were classified according to serum E2 concentrations (<20 ng/ml; 20-45 ng/ml; 45-200 ng/ml; and >200 ng/ml), as shown in Table 1. With the exception of income-to-poverty ratios, there were significant differences in baseline characteristics for different concentrations of E2. Compared with other subgroups, participants with the highest E2 concentrations were more likely to be married, black, and have high total testosterone concentrations and BMI (18-24).

### 3.2. Association between Serum Copper and E2

**Figure 2:**
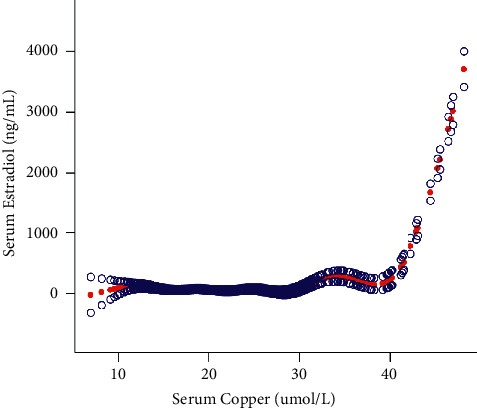
Association between serum copper and serum E2. The solid red line represents the smooth curve fit between variables. Blue bands represent the 95% confidence interval from the fit. Age, race, education, ratio of family income to poverty, testosterone, total, body mass index, and marital status were adjusted.

The results of the multivariate regression analyses are shown in Table 2. In the unadjusted model, copper was positively associated with E2 (Model 1: *β* = 9.485, 95% CI: 7.213-11.758, *P* < 0.00001). This positive association persisted in Models 2 (Model 2: *β* = 9.374, 95% CI: 7.094-11.655, *P* < 0.00001) and 3 after adjusting for confounders (Model 3: *β* = 8.428, 95% CI: 6.100-10.756, *P* < 0.00001). In the subgroup analyses stratified by sex and race/ethnicity, and reported in Table 2, there was a positive correlation between serum copper and serum E2 in women aged 25-55 years (*β* = 14.160, 95% CI: 9.388-18.932, *P* < 0.00001), over 56 years of age (*β* = 0.766, 95% CI: 0.378-1.154, *P*=0.00012), in whites (*β* = −2.760, 95% CI: −4.458-1.061, *P*=0.00151), other Hispanic (*β* = 36.573, 95% CI: 23.463-49.683, *P* < 0.00001), blacks (*β* = 14.185, 95% CI: 8.863-19.506, *P* < 0.00001), and Mexican Americans (*β* = 30.185, 95% CI: 20.771-39.440, *P* < 0.00001) but not in other races (including multiracial) or 25-year-old females. Smooth curve fittings and generalized additive models used to characterize the nonlinear relationship between serum copper and serum E2 are shown in Figure 2. The association between the two is an inverted U-shaped curve, with inflection points determined using two piecewise linear regression models of 28.57 *μ*mol/L (Table 3). For copper >28.57 *μ*mol/L, every 1 *μ*mol/L increase in copper was associated with a 52.819 pg/ml increase in E2 (95% CI: 45.911, 59.727); for copper <28.57 *μ*mol/L, every 1 *μ*mol increase in copper/L was associated with a 4.51 pg/ml (95% CI: −7.456, −1.567) decrease in E2.

### 3.3. Association between Serum Selenium and E2

**Figure 3:**
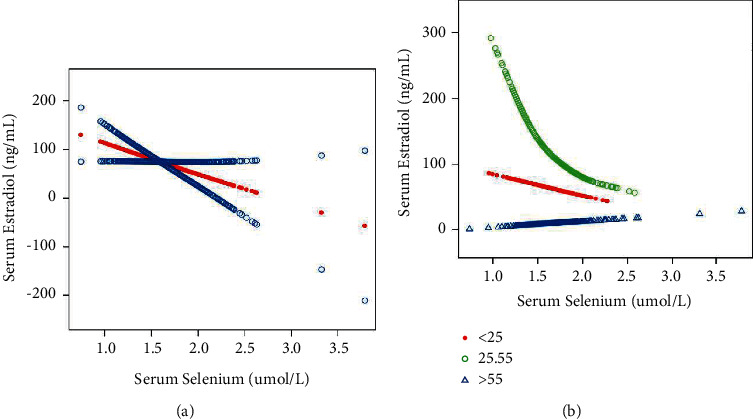
Association between serum selenium and serum E2. (a) No subtype analysis. Age, race, education, ratio of family income to poverty, testosterone, total, body mass index, and marital status were adjusted. (b) Subtype analysis by age. Age was not adjusted, and other adjustments were the same as in (a).

The results of the multivariate regression analyses are shown in Table 4. In the unadjusted model (Model 1: *β* = −92.971, 95% CI: −45.950-39.992, *P* < 0.00059), selenium was highly negatively correlated with E2. This negative correlation persisted in Model 2 (Model 2: *β* = −68.020, 95% CI: −121.724-14.317, *P* < 0.01311) and Model 3 (Model 3: *β* = −56.811, 95% CI: −109.592-7.282, *P* < 0.03499) after adjusting for confounders. In the subgroup analysis stratified by sex and race/ethnicity, and reported in Table 5, serum selenium and serum E2 were negatively correlated in women younger than 25 years (*β* = −62.849, 95% CI: −113.010 to −12.687, *P*=0.01427) and in women aged 25-55 (*β* = −141.971, 95% CI: −273.296-10.647, *P*=0.03437) and in Mexican Americans (*β* = −364.527, 95% CI: −652.132 to −76.922, *P*=0.01334), but not in white, black, other Hispanic, other races (including multiracial), and women over 56 years. Smooth curve fittings and generalized additive models used to characterize the nonlinear relationship between serum selenium and serum E2 are shown in Figure 3. Among women aged 25-55 years, the association between selenium and E2 was an inverted U-shaped curve, with the point of inflection of 1.39 *μ*mol/L identified using a two-piecewise linear regression model. For selenium <1.39 *μ*mol/L, each 1 *μ*mol/L increase in selenium was associated with a 708.164 pg/ml decrease in E2 (95% CI: (−1304.787-111.540); in contrast, for selenium>1.39 *μ*mol/L in individuals, a 1 *μ*mol/L increase in selenium was associated with a 44.255 pg/ml decrease in E2 (95% CI: −199.963-111.453).

### 3.4. Associations of Serum Zinc with E2

The results of the multivariate regression analyses are shown in Table 6. In the unadjusted model, zinc was inversely associated with E2 (Model 1: *β* = −5.953, 95% CI: −11.361 to −0.546, *P* < 0.03104). This negative correlation persisted in Model 2 (Model 1: *β* = −5.509, 95% CI: −10.884 to −0.134, *P* < 0.04465) after adjusting for confounders. Model 3 found no correlation between the two (Model 1: *β* = −4.727, 95% CI: −10.022-0.568, *P* < 0.08028).

## 4. Discussion

E2 is a steroid hormone that plays an important role in regulating the normal menstrual cycle in women, especially during ovarian follicle growth and maturation, and is considered an important marker of follicle quality [16]. Too much E2 can also lead to complications such as endometrial hyperplasia, endometrial cancer [17, 18], and breast cancer [19]. There are many causes of elevated E2, such as polycystic ovary syndrome (PCOS) [20], taking DEHA drugs [21], ovarian tumors [22], and other diseases. Copper, selenium, and zinc are important trace elements for human growth and development.

The correlation between copper, zinc, selenium, and E2 has been studied by many scholars. Sun et al. found that cultured human granulosa cells overexposed to copper exhibited significantly increased E2 secretion and decreased testosterone levels [23]. Wang et al. research team found that selenium treatment also increased the secretion of E2 and P4, stimulated granulosa cell proliferation and steroidogenesis, and reduced apoptosis [24]. Osadchuk et al. research team found that zinc and E2 levels were inversely correlated in men [14].

Our results showed a positive correlation between serum copper levels and serum E2 in women, with the exception of other ethnic groups (including multiracial). The subgroup analysis by ethnicity showed that the serum copper content of women was positively correlated with serum E2 in Mexican American, other Hispanic, and non-Hispanic black populations. In non-Hispanic white individuals, the serum copper content of women was negatively correlated with serum E2. According to age subgroup analysis, it was found that, except for women <25, serum copper levels were positively correlated with serum E2 in women 25-55 and in women over the age of 56 years. The relationship creating the smooth curve fit between female serum copper content and E2 levels was further investigated. It was found to be an inverted U-shaped curve. A bidirectional linear regression model was used to determine the inflection point. The inflection point was 28.57 *μ*mol/L. Previous studies by scholars have reported that copper can promote and increase the secretion of E2 [23]. This study is in line with a previous report. This study further confirms, for the first time, that the inflection point of female serum copper and E2 is 28.57 *μ*mol/L.

Our results showed that serum selenium levels were inversely correlated with serum E2 in women. Subgroup analyses by ethnicity showed that only in Mexican Americans were serum selenium levels negatively correlated with serum E2. Subgroups analyses by age found that the serum selenium content of women was negatively correlated with serum estradiol in women <25 years old and 25-55 years old but not in women >56 years old. Furthermore, in the age subgroups analyses, the relationship creating a smooth curve fit between women's serum selenium content and E2 level was examined, and it was found that women aged 25-55 presented an inverted U-shaped curve. The bidirectional linear regression model was used to determine the inflection point, and the inflection point was found to be 1.39 *μ*mol/L. Previous studies by scholars have reported that serum selenium in women can promote and increase E2 secretion [24]. This study found different results from previous research. Data analyses showed that serum selenium reduced E2 secretion. This study further confirmed for the first time that the inflection point of female serum selenium and E2 is 1.39 *μ*mol/L.

Adjusted for age and race variables, our results showed that serum zinc levels in women were negatively correlated with serum E2, and after further adjustment for age, race, testosterone, and other factors, it was found that serum zinc levels were not correlated with serum E2. Previous scholars believed that zinc plays a very important role in the male reproductive system, and serum zinc was negatively correlated with E2 in this study [14]. The results of this study, unadjusted for variables, were the same as previously reported.

We used a nationally representative sample and performed subgroup analyses. To our knowledge, this is the first study to report the relationship between serum copper, zinc, selenium, and serum E2 and to identify inflection points. However, our study also has limitations, and the cross-sectional design of our study limits the inference of a causal relationship between copper, zinc, selenium, and serum E2 in women. Therefore, further basic mechanistic studies and prospective studies with large samples are warranted to determine the exact mechanism of the association between copper, zinc, selenium, and serum E2 in women.

## 5. Conclusion

Our study demonstrated a positive relationship between serum copper and E2 levels in the female population, with an inverted U-shaped curve (inflection point: 28.57 *μ*mol/L). There was a negative correlation between serum selenium and E2 levels, with an inverted U-shaped curve (inflection point: 1.39 *μ*mol/L). Adjusted for age and race variables, our results showed that serum zinc levels in women were negatively correlated with serum E2, and after further adjustment for age, race, testosterone, and other factors, it was found that serum zinc levels were not correlated with serum E2. In women, measurements of serum copper and selenium may be indirect markers of E2 levels.

## Figures and Tables

**Table 1:** Weighted characteristics of the study population based on serum E2 quartiles.

Estradiol (pg/mL)	<25	25-45	45-200	200	*P -*value
Testosterone, total (ng/dL)	17.888 ± 14.268	25.170 ± 23.385	28.624 ± 22.894	40.036 ± 33.866	<0.00001
Age	51.858 ± 22.206	30.976 ± 14.348	32.960 ± 11.975	32.225 ± 11.217	<0.00001

Race/Hispanic origin					<0.00001
Mexican American	7.811	15.690	13.530	15.400	
Other Hispanic	5.620	8.378	7.384	6.486	
Non-Hispanic white	70.158	52.136	57.650	53.576	
Non-Hispanic black	8.459	13.925	12.619	15.050	
Other Race—Including multiracial	7.952	9.871	8.817	9.489	

education level—adults 20+					<0.00001
Less than 9th grade	5.777	2.950	2.773	5.456	
9-11th grade	6.251	8.646	6.504	7.139	
High school graduate/GED or equivalent	20.324	10.759	14.337	11.472	
Some college or above	52.684	51.275	59.110	58.918	
Missing	14.964	26.370	17.275	17.015	

Marital status					<0.00001
Married	44.249	42.524	52.226	62.639	
Widowed, divorced, or separated	30.171	9.563	9.314	8.963	
Never married	10.617	21.543	21.185	11.383	
Missing	14.964	26.370	17.275	17.015	

Ratio of family income to poverty					0.05412
<1.99	34.835	41.834	38.084	39.034	
1.99-3.49	21.071	24.194	19.852	23.933	
>3.49	36.950	26.479	36.107	32.774	
Missing	7.145	7.492	5.957	4.259	

Body.mass.index					<0.00001
<18.5	7.822	3.465	2.837	1.920	
18.5-24	22.453	20.277	31.524	35.960	
24-28	21.650	22.514	22.703	17.548	
>28	47.204	52.992	42.403	42.511	
Missing	0.871	0.751	0.533	2.062	

Mean ± SD for continuous variables: *P-*value was calculated using a weighted linear regression model. (%) for categorical variables: the *P-*value was calculated using a weighted chi-square test.

**Table 2:** Association between serum copper (*μ*mol/L) and E2 (ng/mL).

	Model 1 *β* (95% CI) *P-*value	Model 2 *β* (95% CI)*P*-value	Model 3 *β* (95% CI) *P*-value
Total	9.485 (7.213, 11.758) <0.00001	9.374 (7.094, 11.655) <0.00001	8.428 (6.100, 10.756) <0.00001

Age			
Total	8.797 (6.525, 11.070) <0.00001	8.618 (6.322, 10.915) <0.00001	8.554 (6.228, 10.880) <0.00001
<25	2.636 (0.603, 4.668) 0.01121	2.237 (0.194, 4.280) 0.03220	1.586 (−0.235, 3.407) 0.08822
25-55	13.846 (9.217, 18.474) <0.00001	13.561 (8.888, 18.234) <0.00001	14.160 (9.388, 18.932) <0.00001
56	0.838 (0.460, 1.217) 0.00002	0.735 (0.344, 1.127) 0.00025	0.766 (0.378, 1.154) 0.00012

Race/Hispanic origin			
Total	12.412 (9.789, 15.036) <0.00001	12.925 (10.300, 15.549) <0.00001	11.643 (8.960, 14.327) <0.00001
Mexican American	28.956 (20.355,37.558) <0.00001	31.148 (22.389, 39.908) <0.00001	30.105 (20.771, 39.440) <0.00001
Other Hispanic	39.199 (27.082,51.317) <0.00001	40.041 (27.917, 52.166) <0.00001	36.573 (23.463, 49.683) <0.00001
Non-Hispanic white	−1.920 (−3.724, −0.117) 0.03722	−1.838 (−3.613, −0.063) 0.04267	−2.760 (−4.458, −1.061) 0.00151
Non-Hispanic black	15.202 (9.730, 20.674) <0.00001	16.330 (10.823, 21.836) <0.00001	14.185 (8.863, 19.506) <0.00001
Other race—including multiracial	−0.102 (−2.031, 1.826) 0.91709	−0.010 (−1.914, 1.893) 0.99161	−0.425 (−2.228, 1.378) 0.64431

Model 1: no covariates were adjusted. Model 2: age and race were adjusted. Model 3: age, race, education, ratio of family income to poverty, testosterone, total, body mass index, and marital status were adjusted. In the subgroup analysis stratified by age and race, the model was not adjusted for age and race.

**Table 3:** Threshold effect analysis of serum copper and serum E2 in women using a two-piecewise linear regression model.

Estradiol (pg/mL)	*β* (95% CI) *P-*value
Model 1	
A straight-line effect	8.428 (6.100, 10.756) <0.0001
Model 2	
Inflection point (K)	28.57
Serum copper <28.57 umol/L	−4.512 (−7.456, −1.567) 0.0027
Serum copper >28.57 umol/L	52.819 (45.911, 59.727) <0.0001
Equation predictions at breakpoints	28.139 (0.194, 56.085)
Log likelihood ratio	<0.001

Age, race, education, ratio of family income to poverty, testosterone, total, body mass index, and marital status were adjusted.

**Table 4:** Association between serum selenium (*μ*mol/L) and E2 (ng/mL).

	Model 1 *β* (95% CI) *P*-value	Model 2 *β* (95% CI) *P*-value	Model 3 *β* (95% CI) *P-*value
Total	−92.971 (−145.950, −39.992) 0.00059	−68.020 (−121.724, −14.317) 0.01311	−56.811 (−109.592, −4.030) 0.03499

Age			
Total	−67.795 (−120.985, −14.604) 0.01255	−68.372 (−121.631, −15.112) 0.01193	−60.032 (−112.783, −7.282) 0.02580
<25	−63.838 (−122.896, −4.779) 0.03443	−60.788 (−119.757, −1.819) 0.04367	−62.849 (−113.010, −12.687) 0.01427
25-55	−139.035 (−269.373, −8.697) 0.03682	−148.320 (−279.474, −17.166) 0.02690	−141.971 (−273.296, −10.647) 0.03437
56	−2.150 (−7.463, 3.163) 0.42796	−1.366 (−6.672, 3.939) 0.61395	0.344 (−4.915, 5.603) 0.89803

Race/Hispanic origin			
Total	−89.588 (−142.588, −36.588) 0.00094	−68.020 (−121.724, −14.317) 0.01311	−56.811 (−109.592, −4.030) 0.03499
Mexican American	−339.806 (−619.704, −59.908) 0.01773	−333.846 (−622.363, −45.329) 0.02379	−364.527 (−652.132, −76.922)0.01334
Other Hispanic	−260.025 (−547.437, 27.387) 0.07719	−231.269 (−528.116, 65.579) 0.12780	−258.957 (−563.502, 45.588) 0.09667
Non-Hispanic white	−44.632 (−79.351, −9.913) 0.01194	−25.603 (−59.777, 8.571) 0.14238	−15.821 (−48.087, 16.445) 0.33683
Non-Hispanic black	−55.100 (−211.408, 101.207) 0.48997	−31.168 (−187.715, 125.379) 0.69655	−61.797 (−210.473, 86.878) 0.41571
Other race—including multiracial	−78.140 (−122.419, −33.861) 0.00061	−52.470 (−99.049, −5.891) 0.02790	−9.188 (−51.084, 32.707) 0.66757

Model 1: no covariates were adjusted. Model 2: age and race were adjusted. Model 3: age, race, education, ratio of family income to poverty, testosterone, total, body mass index, and marital status were adjusted. In the subgroup analysis stratified by age and race, the model was not adjusted for age and race.

**Table 5:** Threshold effect analysis of serum selenium and serum E2 in women using a two-piecewise linear regression model.

Estradiol (pg/mL)	*β* (95% CI) *P-*value
20-55 year-old female	
Fitting by the standard linear model	−127.949 (−259.289, 3.391) 0.0565
Fitting by the two-piecewise linear model	
Inflection point (K)	1.39
Serum selenium <1.39 umol/L	−708.164 (−1304.787, −111.540) 0.0202
Serum selenium >1.39 umol/L	−44.255 (−199.963, 111.453) 0.5776
Equation predictions at breakpoints	116.283 (70.943, 161.624)
Log likelihood ratio	0.048

Race, education, ratio of family income to poverty, testosterone, total, body mass index, and marital status were adjusted.

**Table 6:** Association between serum zinc (*μ*mol/L) and E2 (ng/mL).

	Model 1 *β* (95% CI) *P*-value	Model 2 *β* (95% CI) *P*-value	Model 3 *β* (95% CI) *P-*value
Total	−5.953 (−11.361, −0.546) 0.03104	−5.509 (−10.884, −0.134) 0.04465	−4.727 (−10.022, 0.568) 0.08028

Model 1: no covariates were adjusted. Model 2: age and race were adjusted. Model 3: age, race, education, ratio of family income to poverty, testosterone, total, body mass index, and marital status were adjusted.

## Data Availability

The survey data are publicly available on the Internet for data users and researchers throughout the world at http://www.cdc.gov/nchs/nhanes.
